# Abnormal body mass index may be related to poor social function of female children by a propensity score matching analysis

**DOI:** 10.1038/s41598-021-85911-1

**Published:** 2021-03-18

**Authors:** You Yang, Zhijuan Jin, Shijian Liu, Xingming Jin, Hong Huang, Shilu Tong

**Affiliations:** 1 Department of Developmental and Behavioral Pediatrics, Shanghai Children’s Medical Center, Shanghai Jiaotong University School of Medicine, 1678 Dongfang Road, Shanghai, 200127 People’s Republic of China; 2 Pediatric Translational Medicine Institute, Shanghai Children’s Medical Center, Shanghai Jiaotong University School of Medicine, Shanghai, People’s Republic of China; 3 Shanghai Pubin Children’s Hospital, Shanghai, 200120 People’s Republic of China; 4 Shanghai Key Laboratory of Children’s Environmental Health, Xinhua Hospital, Shanghai Jiaotong University School of Medicine, 1665 Kongjiang Road, Shanghai, 200092 People’s Republic of China; 5 Department of Clinical Epidemiology and Biostatistics, Shanghai Children’s Medical Center, Shanghai Jiaotong University School of Medicine, Shanghai, People’s Republic of China; 6 School of Public Health, Anhui Medical University, Hefei, People’s Republic of China; 7 School of Public Health and Social Work, Queensland University of Technology, Brisbane, Australia

**Keywords:** Diseases, Health care, Risk factors

## Abstract

This study sought to estimate the association of children’s body mass index (BMI) with their social function in Shanghai China. A large population-based cross-sectional study based on a propensity score matching (PSM) analysis was conducted. BMI was compared according to social communication questionnaire (SCQ) classification, and then SCQ score was compared in terms of BMI grouping before and after PSM. A positive SCQ was considered to indicate poor social communication and a negative SCQ was then supposed to be normal. After 1:3 matching, a total of 7563 children aged 3–12 years were included in analysis. There were statistically significant positive correlation of BMI with SCQ scores for obese females of school age (R^2^ = 0.043, p < 0.001) and negative correlation of these two variables for school-aged females with malnutrition (R^2^ = 0.047, p = 0.027). In conclusion, BMI may be characterized as one of predictive factor for poor social function of these children.

## Introduction

Previous studies have demonstrated the impact of nutrition on children’s cognition^1–4^. One study has shown that malnutrition was associated with increasing developmental deficits including suboptimal cognition, communication, and motor function in children^5^. The relationship between body mass index (BMI) and intelligence underscores the importance of nutrition in children’s cognitive development worldwide^2^. On the other hand, children with severe obesity are more likely to have poor non-verbal intelligence quotient^6^. And the higher rate of perceived expressed emotion, psychopathology and low self-esteem was found among obese adolescents^3^. Also, children with higher general executive functions tend to have lower BMI^7^.

Social cognition is a set of cognitive and emotional abilities applied to social situations. Impairments in social cognition are thought to be a hallmark of difficulties in social interactions^8^. The effects of demographic variables on social communication skills have been documented, including child gender^9^, ethnicity^10^, lower family income, maternal education^11^ and electronic screen media (ESM)^12^. However, few studies have examined the relationship between BMI and social skills and the mechanism of this association in children is less understood. In the present study, we set out to determine whether BMI may contribute to children’s social function.

## Methods

### Study population and sampling

**Figure 1:**
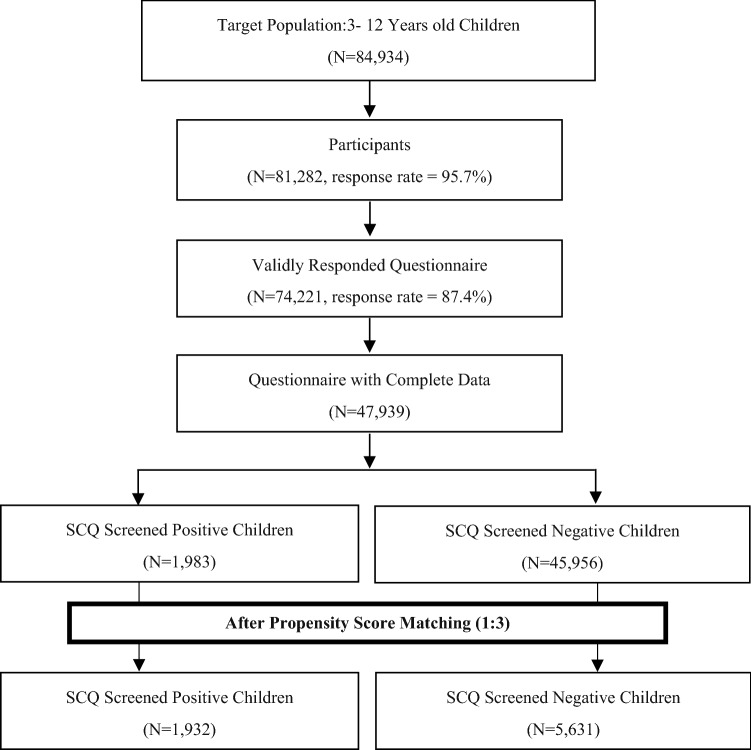
Screening process according to SCQ by parents in Shanghai.

A stratified, random cluster sampling method was used to select 81,282 children aged 3–12 years from kindergartens and primary schools in Shanghai (Fig. 1). The sampling method was as follows: Children from regular schools were randomly sampled from the entire population and seven districts including three urban districts and four suburb districts were randomly selected from seventeen districts in Shanghai. In each selected district, fifteen percent of kindergartens and primary schools were randomly selected. We surveyed all children from special education schools in the seven districts. Special education school services are mainly for children with moderate to severe mental retardation, autism, or multiple disabilities. Children from special education schools were selected in our study because these children are at high risk of poor social communication. Finally, 96 kindergartens, 55 primary schools and 28 special education schools participated in this study. We interviewed all children at their schools during July–August 2014.

### Data collection

Parents and caregivers of selected children were asked to complete the social communication questionnaire (SCQ). The SCQ, which has been standardized in Chinese^13^, was used for measuring children’s social communication skills in this study. The SCQ Current can be used to screen for autism in children aged 2–18 years^14^. The optimum cut-off score for the SCQ is 15. A positive SCQ, i.e. a score equal to or greater than 15 points, was considered to indicate poor social communication. Children with a negative SCQ of less than 15 points were then selected as controls.

Questionnaires were also used for collecting information on the perinatal and gestation variables, children’s families and social environments. Parents or caregivers provided the following information about their children: age, gender, birthweight, education level of parents and caregivers, parents’ personality (introverted or withdrawn/optimistic or open), caregivers, upbringing style, income, history of perinatal period, height, weight, mood, sleep status, outdoor activities, appetite, snack and ESM (daily television viewing time and weekly time spent online or playing video games). Neonatal characteristics including gestational weeks (< 37 weeks, 37–42 weeks and ≥ 42 weeks), normal delivery (yes or no), maternal history of miscarriage (yes or no), early threatening miscarriage (small amount of vaginal bleeding before 28 weeks of pregnancy, yes or no), severe pregnancy reaction (frequent nausea and vomiting, yes or no), asphyxia (lack of oxygen at birth, yes or no), infant feeding patterns (breastfeeding exclusively, formula feeding exclusively, and mixed feeding), parental alcohol drinking and smoking status were considered as potential prenatal confounding factors. Parental socioeconomic characteristics were considered as follows: family income was divided into eight categories (from < 4883.77 U.S. dollars per year to > 32,558.44 U.S. dollars per year), parental education (low: illiterate and primary school; middle: junior school, high school and technical school; high: undergraduate, master and doctor). Bodyweight and height obtained from reported questionnaires were converted into BMI as weight per height squared (kg/m^2^). Normal children (percent 3 ≤ BMI ≤ percent 85), children with malnutrition (BMI < percent 3), overweight (percent 85 < BMI ≤ percent 95) and obesity (BMI > percent 95) were defined according to BMI by the standard of Chinese children^15^. All questionnaires were handed out to the parents or caregivers of the children after the field training.

### Statistical analysis

Variables that showed little variability with a category of the variable containing 95% or more of the observations were not carried forward in the analysis. We used complete data and there was no imputation of data to replace missing observations. Chi-squared tests were used to assess the differences of confounders between SCQ positive and SCQ negative group before and after PSM. Student t-tests were used to assess the differences of BMI and SCQ score after PSM. The curve estimation between BMI and SCQ score was displayed by linear correlation after PSM. All analyses were conducted using IBM SPSS Version 22.0 (IBM Corp., Armonk, NY, USA). Two-tailed and p values of ≤ 0.05 were considered statistically significant.

PSM is a balancing approach whereby a numerical value is assigned for the probability of an intervention. To minimize selection bias inherent in treatment group allocation, PSM was used to match the two groups using a logistic regression approach^16^. An absolute standard bias measure < 0.20 is considered small, and sufficient overlap is required for the propensity scores. In our investigation, we standardized the groups based on propensity. Forty-four covariates were selected including 10 perinatal variables, 9 gestation variables and 25 socioeconomic variables described above.

### Ethics approval and consent to participate

This study was approved by the institutional review boards of the Shanghai Municipal Commission of Health and Family Planning and the Ethics Committee of Shanghai Children’s Medical Center.

## Results

Totally, 81,282 (of 84,934) parents participated in this study with a response rate of 95.7%. Invalid questionnaires were excluded, such as those out of age range and those with more than 30% of information missing. Participants whose answers were inconsistent were also excluded. Hence, the final sample consisted of 47,939 children with complete data regarding all variables.

Among the 47,939 children aged 3–12 years who were included in our analysis, 22,706 (47.32%) were girls and 25,233 (52.68%) were boys. Before matching, children in the SCQ positive group had a mean age of 7.91 years (± 2.56), while controls had a mean age of 7.54 years (± 2.31). Sample characteristics are reported in Supplementary Table S1 (perinatal variables), Supplementary Table S2 (gestation variables) and Supplementary Table S3 (socioeconomic variables).

Distribution of covariates was adequately balanced in the matched data set. After 1:3 matching, a total of 7563 children were included in further analysis, SCQ positive (n = 1932) and SCQ negative (n = 5631). After PSM, mean age was 7.86 (± 2.56) years for SCQ positive group and 7.82 (± 2.31) years for SCQ negative group. We compared the BMI of children between SCQ positive and negative group. A statistically significant difference of BMI between two SCQ groups was observed in girls aged 6–12 years (SCQ positive 17.57 ± 4.85 VS. SCQ negative 16.73 ± 3.65, p < 0.001) after PSM. No statistical difference was obtained in boys aged 3–12 years and girls aged 3–5 years for the difference of BMI between two groups. Comparisons of BMI according to SCQ classification were shown in Table 1.

**Table 1:** Comparison between negative and positive social communication questionnaire (SCQ) with respect to BMI (mean ± standard deviations) before and after propensity score matching (PSM).

		Boys		Girls	
		Aged 3–5	Aged 6–12	Aged 3–5	Aged 6–12
Before PSM	SCQ negative	16.54 ± 3.41	17.72 ± 3.81	16.09 ± 3.50	16.65 ± 3.45
	SCQ positive	16.75 ± 3.56	18.00 ± 4.41	16.13 ± 3.95	17.57 ± 4.84
	*p-*Value	0.218	0.030*	0.891	< 0.001*
After PSM	SCQ negative	16.52 ± 3.40	17.71 ± 3.88	16.06 ± 3.77	16.73 ± 3.65
	SCQ positive	16.78 ± 3.55	17.97 ± 4.32	16.13 ± 3.96	17.57 ± 4.85
	*p-*Value	0.178	0.091	0.850	< 0.001*

*Significant at 0.05 and p-value for t-test.

Similarly, after PSM, SCQ score was high in obese children (BMI > percent 95) compared with that in normal children (percent 3 ≤ BMI ≤ percent 85) for girls aged 6–12 years. For girls aged 6–12 years, there was statistically significant difference between children with malnutrition (BMI < percent 3) and normal children. No statistically significant difference was observed in terms of SCQ score between the normal BMI group and abnormal BMI group (BMI < percent 3 and BMI > percent 95) for boys aged 3–12 years. There was no statistically significant difference in SCQ scores between children with normal BMI and those with overweight (percent 85 < BMI ≤ percent 95). Comparisons according to BMI grouping are detailed in Table 2.

**Table 2:** Comparison between normal BMI (percent 3 ≤ BMI ≤ percent 85) and abnormal BMI (BMI < percent 3 or BMI > percent 95) with respect to SCQ scores (mean ± standard deviations) before and after propensity score matching (PSM).

			Before PSM		After PSM	
			SCQ scores	p value	SCQ scores	p value
Boys	Aged 3–5	Percent 3-percent 85	8.80 + 3.60		11.18 + 4.69	
		< Percent 3	8.69 + 3.70	0.589	10.64 + 4.40	0.343
		> Percent 95	8.83 + 3.79	0.746	11.58 + 4.99	0.209
	Aged 6–12	Percent 3-percent 85	7.69 + 4.02		10.14 + 5.04	
		< Percent 3	8.44 + 4.17	< 0.001*	10.40 + 5.18	0.473
		> Percent 95	7.93 + 4.00	0.003*	10.46 + 5.03	0.134
Girls	Aged 3–5	Percent 3-percent 85	7.67 + 3.29		10.11 + 4.45	
		< Percent 3	8.24 + 3.47	0.003*	11.50 + 4.64	0.051
		> Percent 95	7.91 + 3.23	0.028*	10.17 + 4.07	0.896
	Aged 6–12	Percent 3-percent 85	6.58 + 3.75		9.79 + 5.24	
		< Percent 3	7.15 + 4.15	< 0.001*	11.04 + 5.37	0.020*
		> Percent 95	7.03 + 3.94	< 0.001*	10.69 + 5.34	0.009*

*Significant at 0.05 and p-value for t-test.

**Figure 2:**
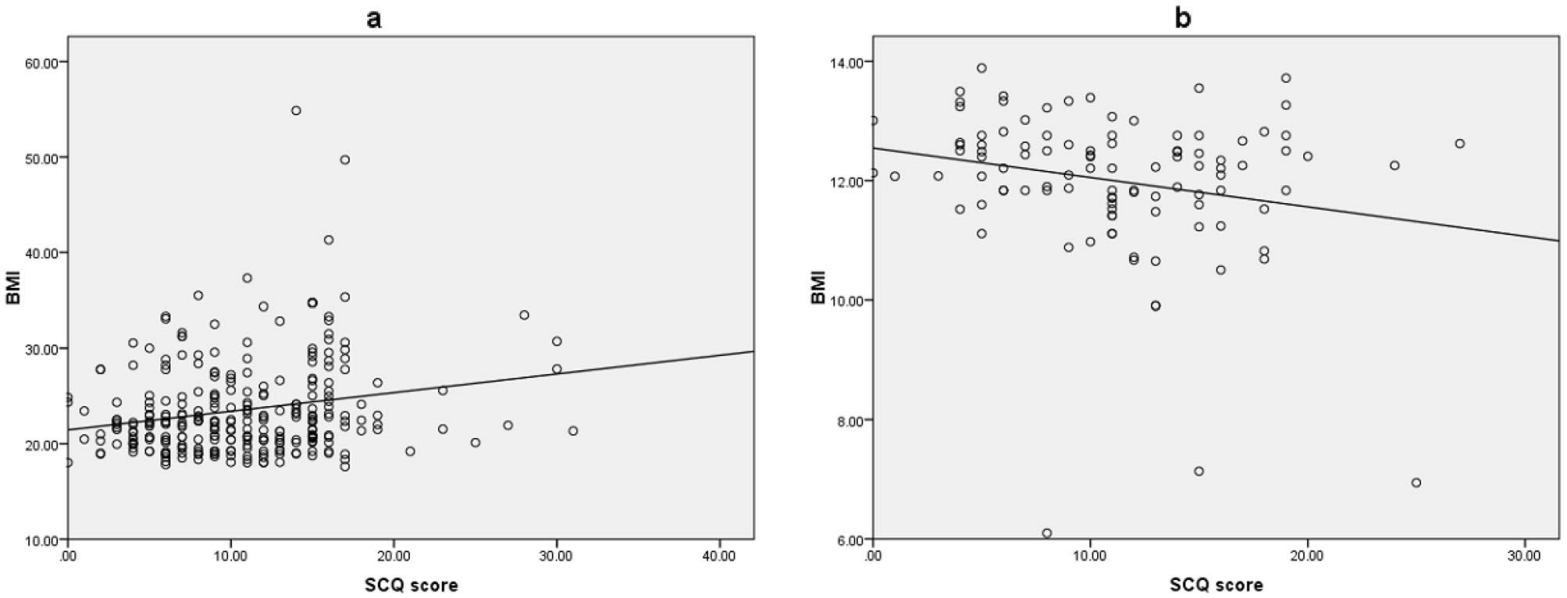
Linear correlation between BMI and SCQ scores for females aged 6–12 years with obesity and malnutrition. (**a**) Illustrates the statistically significant positive correlation of BMI with SCQ scores for obese females aged 6–12 years (R^2^ = 0.043, p < 0.001). (**b**) Displays the statistically significant negative correlation of the two variables for females aged 6–12 years with malnutrition (R^2^ = 0.047, p = 0.027).

Figure 2a illustrates the statistically significant positive correlation of BMI with SCQ scores for obese girls aged 6–12 years (R^2^ = 0.043, p < 0.001). Figure 2b displays the statistically significant negative correlation of the two variables for girls aged 6–12 years with malnutrition (R^2^ = 0.047, p = 0.027).

## Discussion

Multiple studies indicated the social consequences of suffering overweight with social exclusion or isolation^17–19^. In our study, the SCQ was used to evaluate the possible effect of abnormal BMI on social communication. The SCQ performed well with a cut-off of 15 for discriminating between autism and non-autism diagnosis^20^. Also the sensitivity–specificity balance was better in a general population comparing children with autism to typically developing children^21^. This study provides the evidence of a gender difference for the association of BMI with SCQ. Overweight female children showed increasing levels of depressive symptoms^22^ and depressed people usually avoid social situations^23^. According to our cross-sectional study, attention should also be paid to high BMI for girls with poor social function. This gender difference may be related to their physical self-perception. In a recent study, boys showed better physical self-perception than girls in all subscales of physical self-perception profile and girls are at risk for their low physical self-confidence with their respective insecurity feelings and psychological disorders^24^. Social norms may also contribute to the positive correlation between BMI and SCQ scores for girls. In some developing countries, anti-fat norms may particularly concern females and pro-fat norms might persist among males^25,26^.

We also found a link between malnutrition (BMI < percent 3) and SCQ. Although, accumulating evidence suggests that early malnutrition is associated with long-term deficits in cognitive delay^1^, most of previous studies focus on the adverse effects of obesity and there are few reports about the association of social function with malnutrition in school aged children. In one study, children with malnutrition had low social quotient as compared to those with normal nutrition^27^. An animal study indicated that litters of the low protein diet group had lesser weight gain during lactation and showed impaired social discrimination abilities in the homing behavior test^28^. Our study provides a supportive note to this association and further demonstrates that malnutrition in female children of school age is correlated with poor SCQ status, as seen in Fig. 2b. Thus, BMI should be considered as an important physical index to provide suitable social function in children and adolescents, especially for girls.

A strength of our study was the homogeneity of the large sample and that a multilevel analysis was conducted for age, sex and classification of BMI and SCQ with matching for a large number of potentially confounding variables. It is common to use logistic regression analysis in most of previous research. However, many correlated confounders are difficult to control and distinguish, which may lead to discrepancies due to mismanagement of collinearity. Therefore, in this research, we conducted PSM module to balance the bias resulting from confounders. And the results should be more reliable and reproducible.

This study has several limitations, including the self-reported information and cross-sectional analysis. Moreover, the intrinsic limits of the propensity score method may not be negligible. Indeed, propensity score can balance variables identified in the analysis, but it does nothing to balance unmeasured confounders. In our study, we matched a total of 44 variables, which are expected to reduce this impact.

## Conclusion

In summary, the correlation of adverse BMI (both obesity and malnutrition) with poor social skills were observed for school aged female children in our study. BMI may be characterized as one of predictive factor for poor social function of these children. Based on our findings, greater efforts are needed to develop early interventions to reduce weight gain and malnutrition in this pediatric population for their potential impairment in social communication.

## Supplementary Information

Supplementary Information.
