# Supplementary material for: Abnormal body mass index may be related to poor social function of female children by a propensity score matching analysis
Source: Sci Rep. 2021 Mar 18;11:6333. doi: 10.1038/s41598-021-85911-1 (PMC7973791; doi:10.1038/s41598-021-85911-1)
Supplement: Supplementary file 1 — Supplementary Information. [file 41598_2021_85911_MOESM1_ESM.pdf]

Supplementary Table S1 Comparison between negative and positive SCQ with respect to perinatal variables before and after PSM

	Before PSM			After PSM		
	Negative n (%) or mean (± SD)[a]	Positive n (%) or mean (± SD)[a]	*p*-Value	Negative n (%) or mean (± SD)[a]	Positive n (%) or mean (± SD)[a]	*p*-Value, *t*-test [a]
Number of Participants	45986	1983		5631	1932	
Gender			<0.001*			0.557
Female	22088(97.3)	618(2.7)		1752(74)	615(26)	
Male	23868(94.6)	1365(5.4)		3879(74.7)	1317(25.3)	
Age (years)	7.54+2.31 [a]	7.91+2.56 [a]	<0.001* [a]	7.83+2.31 [a]	7.87+2.56 [a]	0.526
Gestational period (weeks)			<0.001*			0.893
⩾37 and <42	41976(96.1)	1725(3.9)		4917(74.4)	1690(25.6)	
<37	2379(93.7)	159(6.3)		437(74.2)	152(25.8)	
⩾42	1601(94.2)	99(5.8)		277(75.5)	90(24.5)	
Birth weight (g)			<0.001*			0.941
2,500–4,000	39481(96)	1641(4)		4700(74.5)	1606(25.5)	
<2,500	1716(93.2)	126(6.8)		327(74)	115(26)	
>4,000	4759(95.7)	216(4.3)		604(74.1)	211(25.9)	
Type of delivery			<0.001*			0.808
Vaginal	20770(95.3)	1019(4.7)		2847(74.3)	983(25.7)	
Caesarian	25186(96.3)	964(3.7)		2784(74.6)	949(25.4)	
History of miscarriage			0.079			0610
Yes	11412(96.1)	458(3.9)		1332(74.9)	446(25.1)	
No	34544(95.8)	1525(4.2)		4299(74.3)	1486(25.7)	
Feeding pattern			<0.001*			0.846
Breastfeeding	21941(95.6)	1006(4.4)		2867(74.7)	970(25.3)	
Formula feeding	7024(95.4)	335(4.6)		953(74.4)	328(25.6)	
Mixed feeding	16991(96.4)	642(3.6)		1811(74.1)	634(25.9)	
Neonatal jaundice			0.150			0.361
Yes	3483(96.3)	133(3.7)		417(76.1)	131(23.9)	
No	42473(95.8)	1850(4.2)		5214(74.3)	1801(25.7)	
Number of siblings			<0.001*			0.924
0	34692(96.5)	1273(3.5)		3666(74.6)	1248(25.4)	
1	4798(95.5)	228(4.5)		677(75)	226(25)	
2	5450(93.6)	374(6.4)		1022(74.1)	358(25.9)	
3	884(90.9)	89(9.1)		221(72.7)	83(27.3)	
4 or above	132(87.4)	19(12.6)		45(72.6)	17(27.4)	
Birth order			<0.001*			0.820
First	40171(96.2)	1592(3.8)		4542(74.4)	1560(25.6)	
Second	5195(94.2)	321(5.8)		921(74.9)	308(25.1)	
Third	524(89.9)	59(10.1)		146(73)	54(27)	
Fourth or above	66(85.7)	11(14.3)		22(68.8)	10(31.3)	

[a] Means and standard deviations and p-value for t-test for continuous variables; *Significant at 0.05; g=grams;

SCQ=social communication questionnaire; PSM=propensity score matching

Supplementary Table S2 Comparison between negative and positive SCQ with respect to gestation variables before and after PSM

	Before PSM			After PSM		
	Negative, n (%)	Positive, n (%)	*p*-Value	Negative, n (%)	Positive, n (%)	*p*-Value
Maternal age at delivery (years)			<0.001*			0.691
25–34	1919(95.6)	88(4.4)		239(73.8)	85(26.2)	
<25	29297(96.4)	1102(3.6)		3202(74.8)	1077(25.2)	
>34	14740(94.9)	793(5.1)		2190(74)	770(26)	
Paternal age at delivery (years)			<0.001*			0.735
25–34	5523(96.2)	217(3.8)		639(75.2)	211(24.8)	
<25	33041(96.1)	1339(3.9)		3832(74.5)	1309(25.5)	
>34	7392(94.5)	427(5.5)		1160(73.8)	412(26.2)	
Paternal alcohol drinking status			0.106			0.803
Drinker	9067(96.2)	362(3.8)		1032(74.2)	359(25.8)	
Non-drinker	36889(95.8)	1621(4.2)		4599(74.5)	1573(25.5)	
Maternal alcohol drinking status			0.661			0.594
Drinker	23106(95.8)	1007(4.2)		2893(74.7)	979(25.3)	
Non-drinker	22850(95.9)	976(4.1)		2738(74.2)	953(25.8)	
Paternal smoking status			<0.001*			0.980
Smoker	23963(95.9)	1028(4.1)		2931(74.5)	1005(25.5)	
Non-smoker	21993(95.8)	955(4.2)		2700(74.4)	927(25.6)	
Psychological state of mother during pregnancy			<0.001*			0.397
Cheerful	42744(96.2)	1700(3.8)		4904(74.6)	1668(25.4)	
Anxious or depressed	3212(91.9)	283(8.1)		727(73.4)	264(26.6)	
Early threatening miscarriage			0.099			0.580
Yes	3224(96.4)	120(3.6)		361(75.5)	117(24.5)	
No	42732(95.8)	1863(4.2)		5270(74.4)	1815(25.6)	
Severe pregnancy reaction (frequent nausea and vomiting)			0.974			0.707
Yes	2796(95.9)	121(4.1)		325(73.7)	116(26.3)	
No	43160(95.9)	1862(4.1)		5306(74.5)	1816(25.5)	
Anemia			0.010*			0.398
Yes	3093(95)	163(5)		427(73)	158(27)	
No	42863(95.9)	1820(4.1)		5204(74.6)	1774(25.4)	

*Significant at 0.05

SCQ=social communication questionnaire; PSM=propensity score matching

Supplementary Table S3 Comparison between negative and positive SCQ with respect to socioeconomic variables before and after PSM

	Before PSM			After PSM		
	Negative, n (%)	Positive, n (%)	*p*-Value	Negative, n (%)	Positive, n (%)	*p*-Value
Father's personality			<0.001*			0.618
Optimistic or open	39527(96.3)	1516(3.7)		4365(74.6)	1487(25.4)	
Introverted or solitary	6429(93.2)	467(6.8)		1266(74)	445(26)	
Mother's personality			<0.001*			0.648
Optimistic or open	42233(96.6)	1487(3.4)		4804(74.5)	1640(25.5)	
Introverted or solitary	3723(89.3)	445(10.7)		827(73.9)	292(26.1)	
Paternal education			<0.001			0.945
Illiteracy and primary education	16442(97.3)	455(2.7)		1339(74.7)	454(25.3)	
Secondary education	19336(96.4)	720(3.6)		2082(74.5)	712(25.5)	
Tertiary education	10178(92.6)	808(7.4)		2210(74.3)	766(25.7)	
Maternal education			<0.001*			0.694
Illiteracy and primary education	14466(97.4)	388(2.6)		1083(73.6)	388(26.4)	
Secondary education	19784(96.5)	713(3.5)		2085(74.8)	702(25.2)	
Tertiary education	11706(93)	882(7)		2463(74.5)	842(25.5)	
Total family income in past year[a] (US$)			<0.001			0.416
0–4,883.77	4379(91.7)	398(8.3)		1018(73)	377(27)	
4,883.77–16,279.22	16542(95.1)	858(4.9)		2502(75.1)	828(24.9)	
16,279.22–32,558.44	16249(97)	505(3)		1439(74)	505(26)	
>32,558.44	8786(97.5)	222(2.5)		672(75.2)	222(24.8)	
Time separated from parents before 3 years of age			<0.001*			0.867
For more than 6 months	5797(94.6)	330(5.4)		879(73.9)	311(26.1)	
For less than 6 months	5000(95.8)	218(4.2)		635(74.8)	214(25.2)	
No	35159(96.1)	1435(3.9)		4117(74.5)	1407(25.5)	
Parenting style			<0.001*			0.671
Indulging or spoiling children	4977(92.8)	388(7.2)		1028(73.3)	374(26.7)	
Maltreatment	1463(92.1)	125(7.9)		330(73.5)	119(26.5)	
Hands-off attitude	1459(91)	144(9)		382(75)	127(25)	
None of above	38057(96.6)	1326(3.4)		3891(74.8)	1312(25.2)	
Grandparents as main caregivers			0.027*			0.323
Yes	21603(96.1)	882(3.9)		2568(75)	856(25)	
No	24353(95.7)	1101(4.3)		3063(74)	1076(26)	
Parents as main caregivers			<0.001*			0.697
Yes	2663(93.1)	198(6.9)		531(73.9)	188(26.1)	
No	43293(96)	1785(4)		5100(74.5)	1744(25.5)	
Babysitters or someone else as main caregiver			0.909			0.400
Yes	44747(95.9)	1930(4.1)		5496(74.5)	1879(25.5)	
No	1209(95.8)	53(4.2)		135(71.8)	53(28.2)	
Health status of main caregivers			<0.001*			0.567

Good	45159(95.9)	1921(4.1)		5476(74.5)	1874(25.5)	
Poor	797(92.8)	62(7.2)		155(72.8)	58(27.2)	
Educational level of main caregivers						
Illiteracy			<0.001*			0.327
Yes	45427(96)	1917(4)		5499(74.5)	1879(25.5)	
No	529(88.9)	66(11.1)		132(71.4)	53(28.6)	
Primary school			<0.001*			0.298
Yes	42515(96.3)	1652(3.7)		4786(74.7)	1623(25.3)	
No	3441(91.2)	331(8.8)		845(73.2)	309(26.8)	
Middle school			<0.001*			0.959
Yes	33697(96.5)	1211(3.5)		3440(74.5)	1179(25.5)	
No	12259(94.1)	772(5.9)		2191(74.4)	753(25.6)	
High school/technical school			0.023*			0.563
Yes	32892(95.7)	1466(4.3)		4127(74.3)	1429(25.7)	
No	13064(96.2)	517(3.8)		1504(74.9)	503(25.1)	
Junior college			<0.001*			0.812
Yes	35763(95.5)	1677(4.5)		4752(74.5)	1626(25.5)	
No	10193(97.1)	306(2.9)		879(74.2)	306(25.8)	
Bachelor degree			<0.001*			0.960
Yes	34239(95.3)	1681(4.7)		4751(74.4)	1631(25.6)	
No	11717(97.5)	302(2.5)		880(74.5)	301(25.5)	
Advanced degree			<0.001*			0.616
Yes	42166(95.7)	1905(4.3)		5418(74.5)	1854(25.5)	
No	3790(98)	78(2)		213(73.2)	78(26.8)	
Time online or playing games, per week			0.003*			0.927
<60 minutes	17264(95.5)	822(4.5)		2313(74.5)	790(25.5)	
60–120 minutes	12463(96.3)	485(3.7)		1350(74)	474(26)	
121–180 minutes	6640(96.3)	258(3.7)		777(75.4)	254(24.6)	
181–240 minutes	3828(96.2)	152(3.8)		448(74.8)	151(25.2)	
241–300 minutes	2453(95.8)	107(4.2)		318(75)	106(25)	
>300 minutes	3308(95.4)	159(4.6)		425(73)	157(27)	
Television viewing time, per day			<0.001*			0.664
<60 minutes	27316(96.7)	941(3.3)		2682(74.2)	932(25.8)	
60–120 minutes	14732(95.2)	736(4.8)		2148(75.1)	712(24.9)	
121–180 minutes	3023(93.3)	217(6.7)		593(74)	208(26)	
>180 minutes	885(90.9)	89(9.1)		208(72.2)	80(27.8)	
Often depressed			<0.001*			0.610
No	37853(96.7)	1308(3.3)		3832(74.7)	1295(25.3)	
Sometimes	6689(92.8)	516(7.2)		1422(74.1)	497(25.9)	
Yes	1414(89.9)	159(10.1)		377(72.9)	140(27.1)	
Appetite			<0.001*			0.806
Very good	10592(96.3)	408(3.7)		1142(73.9)	404(26.1)	
Good	14530(96.5)	524(3.5)		1553(75.2)	512(24.8)	
General	16641(95.7)	745(4.3)		2136(74.6)	726(25.4)	

Poor	3893(93.4)	273(6.6)		717(73.3)	261(26.7)	
Very poor	300(90.1)	33(9.9)		83(74.1)	29(25.9)	
Regular snacks			<0.001*			0.903
Always	554(91.1)	54(8.9)		140(73.3)	51(26.7)	
Often	12407(95)	650(5)		1808(74.1)	633(25.9)	
Sometime	22950(96.3)	887(3.7)		2540(74.5)	870(25.5)	
Seldom	9801(96.3)	378(3.7)		1096(75)	365(25)	
Never	244(94.6)	14(5.4)		47(78.3)	13(21.7)	
Sleep status			<0.001*			0.515
Very good	17740(96.6)	623(3.4)		1815(74.9)	608(25.1)	
Good	19958(96)	832(4)		2385(74.5)	816(25.5)	
General	7045(94.4)	420(5.6)		1153(74.1)	404(25.9)	
Poor	1167(92.4)	96(7.6)		263(73.7)	94(26.3)	
Very poor	46(79.3)	12(20.7)		15(60)	10(40)	
Outdoor activities			<0.001*			0.121
Always	5034(97.4)	133(2.6)		379(74)	133(26)	
Often	24161(97)	743(3)		2226(75.1)	740(24.9)	
Sometime	14306(94.7)	793(5.3)		2322(74.9)	777(25.1)	
Seldom	2455(88.7)	314(11.3)		704(71.4)	282(28.6)	

*Significant at 0.05; SCQ=social communication questionnaire; PSM=propensity score matching
